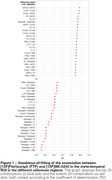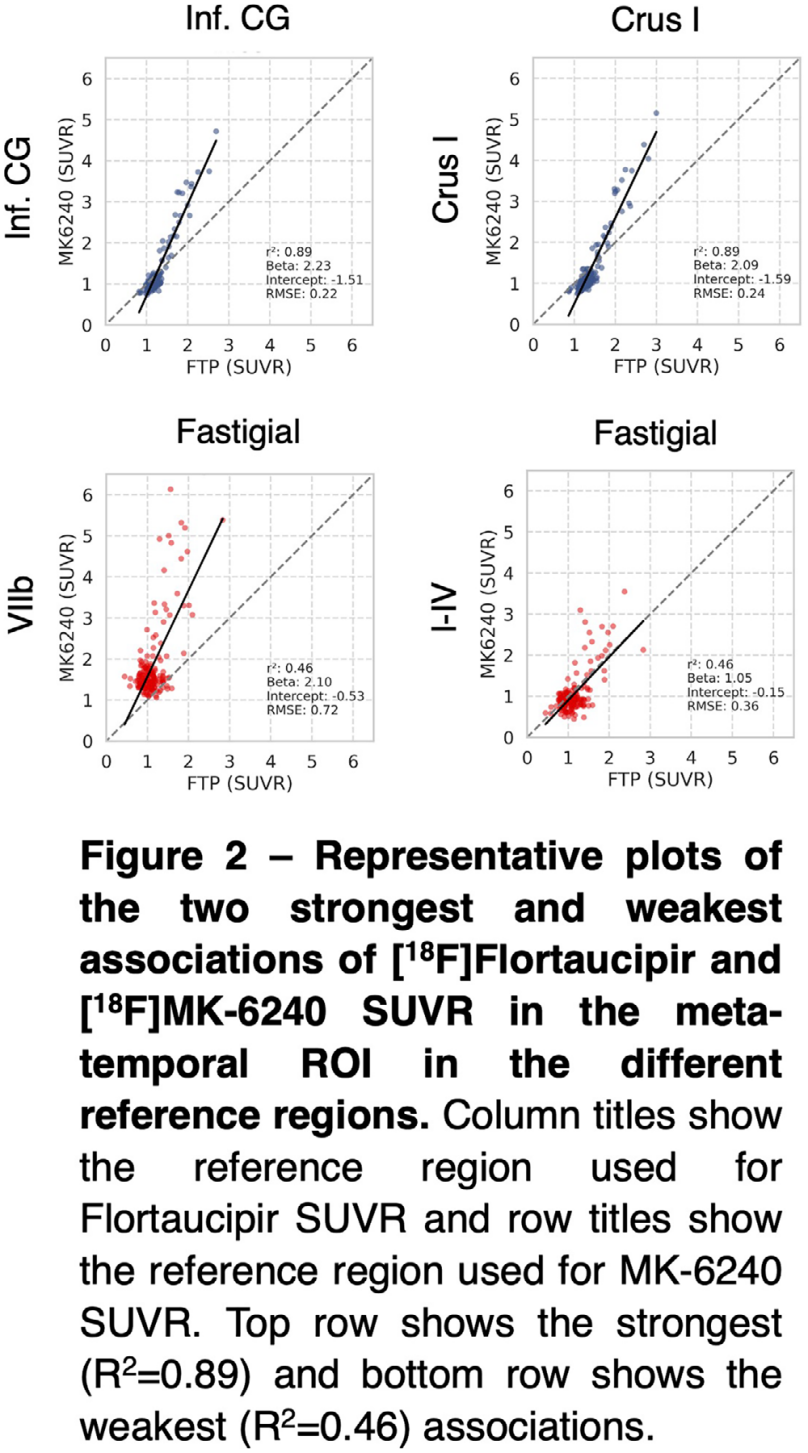# Exploring the effects of using multiple different cerebellar reference regions to improve tau‐PET harmonization ‐ The HEAD Study

**DOI:** 10.1002/alz.094084

**Published:** 2025-01-09

**Authors:** Guilherme Bauer‐Negrini, Guilherme Povala, Bruna Bellaver, Firoza Z Lussier, Cécile Tissot, Hsin‐Yeh Tsai, Livia Amaral, Pamela C.L. Ferreira, Dana Tudorascu, William J. Jagust, William E Klunk, Val J. Lowe, David N. soleimani‐meigooni, Hwamee Oh, Belen Pascual, Brian A. Gordon, Pedro Rosa‐Neto, Suzanne L. Baker, Tharick A. Pascoal

**Affiliations:** ^1^ University of Pittsburgh, Pittsburgh, PA USA; ^2^ Lawrence Berkeley National Laboratory, Berkeley, CA USA; ^3^ Lawrence Berkeley National Lab, Berkeley, CA USA; ^4^ Department of Radiology, Mayo Clinic, Rochester, MN USA; ^5^ Memory and Aging Center, Weill Institute for Neurosciences, University of California, San Francisco, San Francisco, CA USA; ^6^ Brown University, Providence, RI USA; ^7^ Houston Methodist Research Institute, Houston, TX USA; ^8^ Washington University in St. Louis School of Medicine, St. Louis, MO USA; ^9^ Translational Neuroimaging Laboratory, The McGill University Research Centre for Studies in Aging, Montréal, QC Canada

## Abstract

**Background:**

Tau‐PET tracers have been used to diagnose and stage Alzheimer’s disease. However, different tau tracers present distinct patterns of binding throughout the brain, challenging the harmonization of their results. We hypothesize that the choice of a reference region can impact the harmonization of the tau‐PET standardized uptake value ratio (SUVR). In this context, we aimed to explore how different cerebellar reference regions impact the association between [18F]Flortaucipir and [18F]MK‐6240 SUVR values.

**Method:**

We studied 185 individuals across the aging and AD spectrum with head‐to‐head Flortaucipir and MK‐6240 tau PET (HEAD Study). SUVRs were processed to a common 8mm FWHM using 15 different reference regions defined in the spatially unbiased atlas template of the cerebellum (SUIT) (Diedrichsen, 2009). Regression models investigated the association between Flortaucipir and MK‐6240 using multiple combinations of reference regions. R2 statistic was used to estimate goodness‐of‐fitting.

**Result:**

225 combinations of associations Flortaucipir and MK‐6240 SUVR were tested, where the SUVRs are not necessarily quantified using the same reference region. Figure 1 presents the top 25 strongest and 25 weakest associations, ordered based on the coefficient of determination (R²). Flortaucipir and MK‐6240 exhibited the most robust associations when the inferior cerebellar gray matter or Crus I were employed for SUVR determination (R2 = 0.89, Figure 1‐2). Conversely, combinations incorporating the fastigial region consistently yielded weaker associations between the two tracers (R2<0.70, Figure 1‐2).

**Conclusion:**

Interestingly, we showed that the inferior cerebellum, when used for both tracers, serves as a robust reference region for determining the most similar SUVRs for Flortaucipir and MK‐6240. Notably, these two regions have been commonly utilized in previous studies involving these tau tracers. Our results imply that the inferior cerebellum could be the optimal reference region for research aimed at harmonizing tau PET tracers using statistical scales.